# Association of Peripheral Inflammatory Markers with Conception Outcomes Among Women with Endometriosis-Associated Infertility

**DOI:** 10.3390/diagnostics16030462

**Published:** 2026-01-30

**Authors:** Oana Maria Gorun, Adrian Ratiu, Florin Gorun, Cosmin Citu, Voicu Caius Simedrea, Daniela-Eugenia Popescu, Roxana Folescu, Andrei Motoc

**Affiliations:** 1 Doctoral School, “Victor Babes” University of Medicine and Pharmacy Timisoara, Eftimie Murgu Square 2, 300041 Timisoara, Romania; oana-maria.gorun@umft.ro (O.M.G.); caius.simedrea@umft.ro (V.C.S.); 2 Department of Obstetrics and Gynecology, "Victor Babes" University of Medicine and Pharmacy Timisoara, 2 Eftimie Murgu Square, 300041 Timisoara, Romania; citu.ioan@umft.ro (C.C.); popescu.daniela@umft.ro (D.-E.P.); 3 Department of Obstetrics and Gynecology, Municipal Emergency Clinical Hospital Timisoara, 300172 Timisoara, Romania; gorun.florin@umft.ro; 4 Department of Family Medicine, “Victor Babes” University of Medicine and Pharmacy Timisoara, Eftimie Murgu Square 2, 300041 Timisoara, Romania; folescu.roxana@umft.ro; 5 Department of Anatomy and Embryology, “Victor Babes” University of Medicine and Pharmacy Timisoara, Eftimie Murgu Square 2, 300041 Timisoara, Romania; amotoc@umft.ro

**Keywords:** endometriosis, infertility, prognostic biomarkers, systemic inflammation, spontaneous conception

## Abstract

**Background:** Predicting natural conception following surgical treatment remains a clinical challenge for endometriosis, a chronic inflammatory condition often associated with infertility. This study aimed to determine if simple, low-cost inflammatory biomarkers, such as NLR, dNLR, LMR, PLR, SIRI, PIV, and SII calculated from routine preoperative blood tests are associated with spontaneous pregnancy in women with endometriosis-related infertility after laparoscopic surgery. **Methods**: A retrospective analysis was conducted on 78 women between the ages of 18 and 48 who underwent standardized laparoscopic surgery and were monitored for up to 18 months. **Results**: The pregnancy group had significantly lower NLR, dNLR, PLR, SIRI, and especially SII values than the non-pregnancy group. Among the evaluated markers, SII, NLR, and dNLR demonstrated the highest discriminative ability for spontaneous conception. In regression analyses, lower values of NLR and dNLR were associated with higher odds of spontaneous pregnancy. **Conclusions**: These findings suggest a correlation between preoperative inflammatory status and postoperative reproductive outcomes in women with endometriosis.

## 1. Introduction

About 10% of women of reproductive age worldwide are affected by endometriosis, a chronic gynecological disorder characterized by the presence of endometrial-like tissue outside the uterine cavity. Constipation, infertility, dysmenorrhea, and persistent pelvic pain are some of the symptoms linked to this complicated condition [1,2,3,4]. Additionally, 25–50% of women with endometriosis experience infertility, and 35–50% of them have difficulty to conceive [5,6].

Increasing evidence suggests that endometriosis is associated with systemic inflammation, thus the systemic inflammatory response may be associated with changes in different types of white blood cells [7,8,9,10]. Prolonged activation of immune cells, especially neutrophils and macrophages, raises levels of cytokines, interleukins, and tumor necrosis factor, resulting in a proinflammatory environment that may impact reproductive function [11,12,13]. Neutrophil-to-lymphocyte ratio (NLR), derived NLR (dNLR), platelet-to-lymphocyte ratio (PLR), systemic immune-inflammation index (SII), systemic inflammation response index (SIRI), lymphocyte-monocyte ratio (LMR) and pan-immune-inflammation value (PIV) are considered simple, easily available markers reflecting systemic inflammatory status and disease prognosis [14,15,16,17]. Moreover, the preoperative NLR level showed a significant association with the likelihood of achieving spontaneous pregnancy in infertile patients with ovarian endometrioma following surgical treatment [18].

Persistent inflammation associated with endometriosis has been found to have a negative effect on fertility, even after surgical treatment [19]. Systemic activation of the immune system may persist despite laparoscopic excision of endometriotic lesions, affecting ovarian follicle development, endometrial receptivity, and embryo implantation capacity [20].

Therefore, this study aimed to evaluate the association between peripheral inflammatory markers—including NLR, dNLR, PLR, LMR, SIRI, SII, and PIV, and conception outcomes following surgical management in women with endometriosis-associated infertility.

## 2. Materials and Methods

### 2.1. Study Design and Settings

From January 2017 to December 2022, a retrospective observational study was conducted that included women of reproductive age diagnosed with infertility associated with endometriosis who underwent laparoscopic surgery at the Municipal Emergency Clinical Hospital in Timisoara. All patients were between 18 and 48 years of age and had histopathological confirmation of endometriosis after surgery. Male factor infertility had been ruled out prior to inclusion.

Each case was independently reviewed by two investigators to ensure data accuracy and consistency. The study was approved by the Ethics Committee of the University of Medicine and Pharmacy “Victor Babes” Timisoara (Approval No. 22726, 17 November 2021) and conducted in accordance with the STROBE guidelines for observational studies. Informed consent was obtained from all subjects involved in the study.

### 2.2. Participants

Participants were required to have completed preoperative laboratory testing—including a complete blood count performed prior to surgery—and to have documented infertility, defined as failure to conceive after at least 12 months of unprotected intercourse.

*Exclusion criteria* included the presence of:

• Acute infections;
• Hematologic, endocrine, or metabolic disorders;
• Autoimmune or chronic inflammatory diseases;
• Malignancy;
• Ongoing or recent pregnancy;
• Recent use of corticosteroid, hormonal, or immunosuppressive therapy;
• Incomplete medical records;
• Major abdominal or pelvic surgery within the previous six months.

Patients who had undergone assisted reproductive technology (ART), including IVF or IUI, were excluded from the analysis to avoid potential confounding effects related to treatment-specific factors.

Initially, 90 women who were qualified and had endometriosis-related infertility were found. During the follow-up, 12 women could not be reached and were hence eliminated from the analysis. The final group of individuals participating in the study was 78 women, categorized based on the reproductive outcome following surgery:

• Pregnancy group (*n* = 19);
• Non-pregnancy group (*n* = 59).

*Follow-up.* Postoperative reproductive outcomes were assessed through structured telephone follow-up at 12 and 18 months after surgery. When pregnancy was reported, confirmation was obtained from medical records (ultrasound reports or obstetric consultations), whenever available. Patients who could not be contacted after at least three attempts made on different days were considered lost to follow-up.

### 2.3. Variables, Data Sources, and Measurement

Individual patient records were used to verify all medical data that was obtained from the hospital’s electronic database. Age, place of residence, reproductive characteristics (primary or secondary infertility), clinical symptoms (pelvic pain), laboratory results (complete blood count and derived inflammatory indices), surgical results (type and location of endometriotic lesions), and histopathological confirmation of endometriosis were all included in the dataset.

Demographic (age, residence), clinical (type of infertility, pelvic pain), surgical (site and stage of endometriosis), and laboratory (complete blood count and inflammatory indices) variables were all obtained from electronic hospital records using a standardized collection form.

The primary outcome of the study was spontaneous clinical pregnancy, defined as a naturally conceived pregnancy confirmed by ultrasound examination or documented obstetric medical records.

Infertility was defined as the failure to achieve spontaneous conception after at least 12 months of regular unprotected intercourse.

The revised American Society for Reproductive Medicine (rASRM) classification was used to stage endometriosis: Stage I (minimal, 1–5 points), Stage II (mild, 6–15 points), Stage III (moderate, 16–40 points), and Stage IV (severe, >40 points).

The systemic inflammation parameters were determined from complete blood counts collected before surgery using the following formulas: NLR = absolute neutrophil count (ANC)/absolute lymphocyte count (ALC); dNLR = ANC/WBC (white blood cell) − ANC; LMR = ALC/absolute monocyte count (AMC); PLR = absolute platelet count (APC)/ALC; SII = (ANC × APC)/ALC; SIRI = (ANC × AMC)/ALC, and indicate immune regulatory activity. In addition, the systemic immune inflammation index (SII), and pan-immune inflammatory value (PIV) were also determined using the following formulas: SII = platelets count × neutrophil/lymphocyte ratio; PIV = neutrophil count × platelet count × monocyte count/lymphocyte count.

### 2.4. Statistical Analysis

Statistical analysis was performed using SPSS 20.0 software (SPSS Inc., Chicago, IL, USA) and Python 3.10.12 (Beaverton, OR, USA) with standard scientific libraries (pandas, NumPy, SciPy, statsmodels, and scikit-learn). Quantitative variables were tested for normal distribution using the Shapiro–Wilk test and expressed as median (interquartile range). Categorical variables were presented as frequencies and percentages. Comparisons between groups were performed using the Mann–Whitney U test for continuous data and Fisher’s exact test for categorical data.

Receiver Operating Characteristic (ROC) curve analysis was used to evaluate the discriminative ability of inflammatory markers for spontaneous conception after surgery. Optimal cut-offs were calculated for descriptive purposes only. Bootstrap internal validation (2000 resamples) was used to evaluate model optimism, and optimism-corrected AUC values were reported. Using all markers and important covariates, penalized logistic regression (ridge and LASSO) with 5-fold stratified cross-validation (ROC AUC as performance metric) was carried out as a sensitivity analysis.

Statistical significance was defined as *p* < 0.05.

## 3. Results

### 3.1. Baseline Characteristics

Out of the 90 women who were first eligible, 12 were lost to follow-up and were not included. The final study cohort comprised 78 women, with 19 achieving spontaneous clinical pregnancy and 59 not achieving it.

No significant demographic or clinical differences were observed between women who achieved pregnancy after surgery and those who did not (Table 1). Pelvic pain tended to be less frequent among women who conceived (52.6%) compared to those who did not (72.9%), although this difference did not reach statistical significance (*p* = 0.08). Regarding surgical findings, endometriosis location did not differ between groups (*p* = 0.84). Disease severity, assessed using the ASRM score and staging system, showed no significant association with pregnancy outcomes (*p* = 0.55 for score; *p* = 0.38 for stage distribution).

### 3.2. Laboratory Parameters and Inflammatory Markers

Significant differences in preoperative inflammatory markers were observed between the two groups (Table 2). While total white blood cell, monocyte, and platelet counts were comparable between groups, the pregnancy group exhibited higher lymphocyte levels.

Key inflammatory indices—including NLR, dNLR, PLR, SIRI, and especially SII—were significantly lower in women who achieved pregnancy. Markers such as LMR and PIV did not show significant differences (Table 2).

### 3.3. Associations Between Inflammatory Markers and Pregnancy Outcomes

The association between individual hematologic markers and postoperative pregnancy was further explored using univariate logistic regression (Table 3). Conventional blood count parameters such as WBC (white blood cells), neutrophils, monocytes, and platelets were not prognostic correlate of pregnancy. Lymphocyte count showed a borderline association (OR = 2.31; *p* = 0.05).

In contrast, inflammatory ratios exhibited strong prognostic value. Lower NLR significantly increased the likelihood of pregnancy (OR = 0.45; *p* = 0.04), as did lower PLR (OR = 0.98; *p* = 0.03) (Table 3).

### 3.4. Association Between Inflammatory Biomarkers and Spontaneous Conception

**Figure 1:**
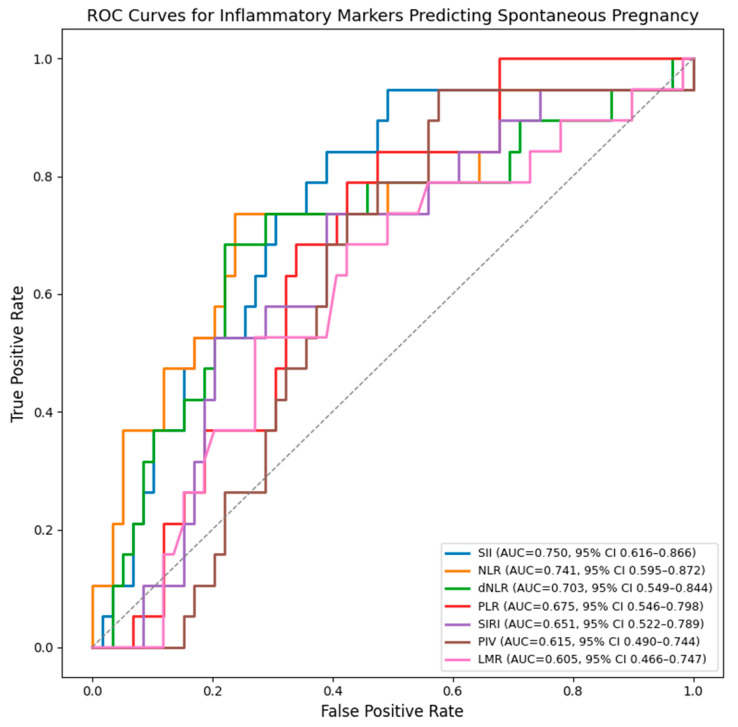
ROC curves for inflammatory markers predicting spontaneous postoperative conception. AUC values were internally validated using bootstrap resampling (1000 iterations) and are presented with 95% confidence intervals.

The ROC (Receiver Operating Characteristic) curves were generated separately for each inflammatory marker curves and show that SII, NLR, and dNLR have the highest discriminative ability for predicting spontaneous pregnancy after surgery. SII demonstrates the best overall performance, followed closely by NLR and dNLR, all with AUC values above 0.70. PLR, LMR, PIV and SIRI show moderate accuracy (Figure 1).

Among the evaluated markers, SII showed the highest discriminative ability for spontaneous pregnancy (AUC 0.750, 95% CI 0.616–0.866), followed by NLR (AUC 0.741, 95% CI 0.595–0.872) and dNLR (AUC 0.703, 95% CI 0.549–0.844). (Table 4).

In parsimonious logistic regression models, inflammatory markers were analyzed as continuous variables (log-transformed and standardized). Separate models were constructed for each marker to avoid multicollinearity, with adjustment limited to age and rASRM stage (III–IV vs. I–II).

Higher NLR and dNLR values were associated with lower odds of spontaneous clinical pregnancy (NLR: OR 0.44 per 1 SD increase in log (NLR), 95% CI 0.23–0.84; dNLR: OR 0.39 per 1 SD increase in log (dNLR), 95% CI 0.17–0.92). Other inflammatory indices were not significantly associated with pregnancy outcome in adjusted models (Table 5).

For parsimonious models that included each marker with age and rASRM stage, bootstrap internal validation (2000 resamples) was carried out. After taking optimism into account, the optimism-corrected AUC for the NLR, dNLR, and SII models was 0.689, 0.704, and 0.620, respectively, indicating moderate discrimination (Table 6).

Ridge regression obtained a cross-validated AUC of roughly 0.69 in sensitivity analyses employing penalized logistic regression. NLR and dNLR remained the only non-zero predictors among inflammatory indices in LASSO regression, which produced lower performance (CV-AUC ≈ 0.59). The type of surgical procedure and the location of endometriosis did not provide any independent predictive information following penalization.

## 4. Discussion

In this study, we assessed the relationship between endometriosis-associated infertility in women and peripheral inflammatory markers and spontaneous clinical pregnancy. Particular focus was placed on reducing overfitting and optimism bias using parsimonious modeling, internal validation, and penalized regression due to the small number of pregnancy events. The strongest discriminative ability was shown by SII, NLR, and dNLR, supporting the growing body of evidence that systemic inflammation plays a critical role in reproductive outcomes in endometriosis-affected women.

According to recent studies, chronic systemic and peritoneal inflammation, which modifies immune cell populations, cytokine secretion, and endometrial receptivity, is a key characteristic of endometriosis [7,21,22]. A comprehensive analysis by Cuadrado-Torroglosa et al. emphasizes that abnormal immunoinflammatory responses interfere with endometrial remodeling, inhibit implantation, and contribute to infertility in women with endometriosis [11]. Moreover, recent investigations have underscored the clinical significance of systemic inflammatory indices obtained from standard blood tests in the assessment of endometriosis, affirming their utility as accessible instruments for risk stratification in inflammation-related disorders [23].

Our findings are also consistent with evidence from Zhou et al., who demonstrated that systemic inflammation markers—SII, SIRI, NLR, and PIV—are significantly elevated in women with endometriosis compared with healthy controls, achieving an AUC of 0.796 when combined [24]. This suggests that these markers, which can be relatively easily calculated from peripheral blood counts, may provide additional value in the evaluation of women with endometriosis-associated infertility [25]. Furthermore, the results of our study complement this evidence, showing that lower preoperative values of these indices—particularly SII and NLR—are associated with spontaneous pregnancy after surgical treatment.

Duan et al., in their research, showed that systemic inflammatory markers (NLR, PLR, SIRI) were independently associated with infertility in a general population, regardless of the underlying cause [26]. This aligns with earlier evidence indicating that NLR is linked to infertility in endometriosis, as demonstrated by Jing et al. [27]. Thus, their findings strengthen the hypothesis that chronic systemic inflammation can affect reproductive capacity in multiple etiologies, including endometriosis.

Additionally, a large study conducted by Chen, using NHANES (National Health and Nutrition Examination Survey) data, provides further support for the importance of systemic inflammation in female fertility. This research analyzed data from 3000 women of childbearing age and concluded that lower levels of SII and PLR were associated with a lower risk of infertility, even after adjusting for major confounding factors [28]. These results are in line with ours, which showed that SII was the best indicator of spontaneous pregnancy following surgery. Although the NHANES study did not specifically focus on endometriosis, the consistent association between lower inflammatory indices and better fertility outcomes in different populations reinforces the relevance of these biomarkers as simple and accessible tools in reproductive health.

In addition to traditional inflammatory markers, there is growing interest in non-invasive biomarkers found in plasma and peritoneal fluid that indicate extracellular matrix remodeling and epithelial–mesenchymal transition, which are crucial to the pathophysiology of endometriosis. Transcription factors like ZEB are involved with epithelial–mesenchymal transition and are suggested as possible indicators for endometriosis-related infertility. These molecular markers may enhance systemic inflammatory indices by offering mechanistic understanding of disease activity and reproductive dysfunction [29]. Along with transcriptional regulators that play a role in epithelial–mesenchymal transition, studies have looked into extracellular matrix–related proteins as non-invasive diagnostics for endometriosis. Proteins like fibronectin and collagen IV, found in plasma and peritoneal fluid, show changes in the extracellular matrix and tissue structure that happen with endometriotic lesions. These markers offer additional mechanistic insights into disease development and reproductive dysfunction, potentially increasing the understanding of endometriosis-related infertility [30].

As strengths, we emphasize that this study addresses an important but insufficiently explored topic: the association between inexpensive and easily accessible inflammatory biomarkers (NLR, dNLR, SII, PLR, LMR, SIRI, PIV) with spontaneous conception after laparoscopic treatment in women with endometriosis-associated infertility. Moreover, the study uses a rigorous analytical framework, including univariate and multivariate logistic regression, to identify independent predictors, supported by ROC curve analysis to evaluate discriminative performance.

This study has some limitations. First, it has a retrospective design. Second, the inflammatory markers were measured only once, before surgery. Also, the number of patients was relatively small, which may limit how well the results apply to larger populations. Residual confounding cannot be ruled out because a number of pertinent confounders, such as ovarian reserve parameters, BMI, smoking status, length of infertility, and postoperative management, were unavailable and could not be included. In order to reduce heterogeneity related to partner-related causes, male factor infertility was systematically excluded before inclusion. Finally, the study was conducted in a single center, so the findings may not reflect all clinical settings.

This investigation was intentionally designed as an exploratory analysis to assess a variety of peripheral inflammatory markers in the context of endometriosis-related infertility. Consequently, the results should be interpreted as a means of generating novel ideas and should be verified in larger, more meticulously designed studies due to the limited number of pregnancy events and the large number of markers that were tested.

## 5. Conclusions

In conclusion, this research shows that inflammatory markers—particularly NLR, dNLR, and SII—are associated with the probability of spontaneous conception after laparoscopic treatment in women with infertility associated with endometriosis. Lower preoperative values of these indices were associated with better reproductive outcomes. Although further research is needed to confirm these findings in larger, prospective cohorts, Inflammatory markers calculated from peripheral blood counts can serve as accessible tools to support postoperative counseling and guide individualized fertility planning in clinical practice.

## Figures and Tables

**Table 1:** Baseline Characteristics among 78 patients with endometriosis associated infertility.

Variable	Total	Pregnancy After Procedure YES (n = 19)	Pregnancy After Procedure NO (n = 59)	*p*-Value
Age	28 (5)	30 (4)	31 (5.5)	0.23 *
Residence Urban	53 (67.9%)	11 (57.9%)	42 (71.2%)	0.39 **
Primary infertility	70 (89.7%)	16 (84.2%)	54 (93.1%)	0.35 **
Presence of pelvic pain	53 (67.9%)	10 (52.6%)	43 (72.9%)	0.08 **
**Endometriosis Site**				
Ovary	34 (43.6%)	9 (47.4%)	25 (42.4%)	0.84 ***
Superficial	6 (7.7%)	2 (10.5%)	4 (6.8%)	
Deep	16 (20.5%)	4 (21.1%)	12 (20.3%)	
Ovary and Deep	15 (19.2%)	2 (21.1%)	13 (20.3%)	
Ovary and superficial	7 (9.0%)	2 (10.5%)	5 (5.5%)	
**Endometriosis Classification**				
ASRM score	4 (20)	8 (12)	12 (20)	0.55 *
ASRM Stage I	21 (26.9%)	5 (26.3%)	16 (27.1)	0.38 ***
ASRM Stage II	26 (33.3%)	9 (47.4%)	17 (28.8%)	
ASRM Stage III	24 (30.8%)	4 (21.1%)	20 (33.9%)	
ASRM Stage IV	7 (9.0%)	1 (5.3%)	6 (10.2%)	

* Calculated by Mann–Whitney U test; ** Calculated by Fisher Exact test; *** Calculated by Fisher’s exact test (Freeman–Halton extension); ASRM = American Society for Reproductive Medicine.

**Table 2:** Comparison of hematologic and inflammatory markers between pregnancy and non-pregnancy groups.

Variable	Pregnancy After Procedure YES	Pregnancy After Procedure NO	*p*-Value
WBC (×109/L)	6.8	6.5	0.39
Neutrophils (×109/L)	3.77	4.4	0.13
Lymphocytes (×109/L)	2.12	1.72	0.03
Monocytes (×109/L)	0.4	0.42	0.72
Platelets (×109/L)	273	297	0.26
NLR	1.87	2.38	0.002
dNLR	1.19	1.88	0.008
PLR	128	172	0.02
LMR	5.74	4.46	0.17
SIRI	0.64	0.98	0.05
PIV	60	76	0.12
SII	525	726	0.001

WBC = White Blood Cell; NLR = Neutrophil to Lymphocyte Ratio; dNLR = derived Neutrophil to Lymphocyte Ratio; PLR = Platelet to Lymphocyte Ratio; LMR = Lymphocyte to Monocyte Ratio; SIRI = Systemic Inflammation Response Index; PIV = Pan Immune Inflammation Value; SII = Systemic Immune-Inflammation Index.

**Table 3:** Univariate logistic regression analysis of preoperative hematologic and inflammatory markers associated with postoperative spontaneous pregnancy.

Variable	B	SE	OR	*p*-Value	95% CI	
					Lower	Upper
WBC (×109/L)	0.129	0.134	1.13	0.33	0.875	1.480
Neutrophils (×109/L)	−0.130	0.167	0.87	0.43	0.632	1.218
Lymphocytes (×109/L)	0.839	0.436	2.31	0.05	0.984	5.446
Monocytes (×109/L)	0.279	1.370	1.32	0.83	0.090	19.388
Platelets (×109/L)	−0.003	0.004	0.99	0.46	0.990	1.005
NLR	−0.796	0.396	0.45	0.04	0.208	0.980
dNLR	−0.076	0.111	0.92	0.49	0.746	1.152
PLR	−0.011	0.005	0.98	0.03	0.979	0.999
LMR	−0.032	0.040	0.96	0.41	0.895	1.047
SIRI	−0.070	0.302	0.93	0.81	0.516	1.684
PIV	−0.004	0.005	0.99	0.45	0.986	1.006
SII	−0.002	0.001	0.99	0.08	0.996	1.000

WBC = White Blood Cells; NLR = Neutrophil to Lymphocyte Ratio; Dnlr = derived Neutrophil to Lymphocyte Ratio; PLR = Platelet to Lymphocyte Ratio; LMR = Lymphocyte to Monocyte Ratio; SIRI = Systemic Inflammation Response Index; PIV = Pan Immune Inflammation Value; SII = Systemic Immune-Inflammation Index; B = B−Coefficient; SE = Standard Error; OR = Odds Ratio; CI = Confidence Interval.

**Table 4:** ROC curve analysis and optimal cut-off values of inflammatory markers for predicting spontaneous pregnancy.

Variable	AUC	95% CI	*p*-Value	Exploratory Optimal Cut-off (Youden Index)	Sensitivity	Specificity
NLR	0.741	0.595–0.872	0.002	2.17	0.76	0.73
dNLR	0.703	0.549–0.844	0.008	1.37	0.77	0.68
PLR	0.675	0.546–0.798	0.024	171.8	0.52	0.84
LMR	0.605	0.466–0.747	0.171	10.0	0.11	1.0
SIRI	0.651	0.522–0.789	0.050	0.87	0.61	0.73
PIV	0.615	0.490–0.744	0.126	94.9	0.42	0.94
SII	0.750	0.616–0.866	0.001	726.6	0.50	0.94

NLR = Neutrophil to Lymphocyte Ratio; Dnlr = derived Neutrophil to Lymphocyte Ratio; PLR = Platelet to Lymphocyte Ratio; LMR = Lymphocyte to Monocyte Ratio; SIRI = Systemic Inflammation Response Index; PIV = Pan Immune Inflammation Value; SII = Systemic Immune-Inflammation Index; AUC = Area Under the Curve; SE = Standard Error.

**Table 5:** Logistic regression models for determining the association between continuous inflammatory markers and spontaneous clinical pregnancy.

Marker	Predictor Scaling	Unadjusted OR	Unadjusted 95% CI	Unadjusted *p*	Adjusted OR *	Adjusted 95% CI	Adjusted *p*
NLR	per 1 SD increase in log(marker)	0.41	0.22–0.78	0.006	0.44	0.23–0.84	0.01
dNLR	per 1 SD increase in log(marker)	0.36	0.16–0.86	0.02	0.39	0.17–0.92	0.03
SII	per 1 SD increase in log(marker)	0.70	0.42–1.20	0.19	0.74	0.44–1.24	0.25
PLR	per 1 SD increase in log(marker)	0.82	0.51–1.31	0.419	0.83	0.52–1.35	0.46
SIRI	per 1 SD increase in log(marker)	0.86	0.52–1.43	0.57	0.84	0.50–1.46	0.55
LMR	per 1 SD increase in log(marker)	1.00	0.60–1.69	0.97	1.05	0.60–1.84	0.85

* Predictor scaling indicates that odds ratios are expressed per one standard deviation increase in the log-transformed inflammatory marker values.

**Table 6:** Multivariate logistic regression.

Model.	Apparent AUC	Optimism	Optimism-Corrected AUC
NLR + age + stage	0.73	0.04	0.68
dNLR + age + stage	0.75	0.05	0.70
SII + age + stage	0.67	0.05	0.62

## Data Availability

The data sets used and/or analyzed during the present study are available from the correspondence author on reasonable request.
